# Impact of Methylene Blue on Enhancing the Hydrocarbon Potential of Early Cambrian Khewra Sandstone Formation from the Potwar Basin, Pakistan

**DOI:** 10.1021/acsomega.3c06923

**Published:** 2023-12-01

**Authors:** Muhammad Ali, Abdul Majeed Shar, Nurudeen Yekeen, Hussein Abid, Muhammad Shahzad Kamal, Hussein Hoteit

**Affiliations:** † Physical Science and Engineering Division, King Abdullah University of Science and Technology (KAUST), Thuwal 23955, Saudi Arabia; ‡ Department of Petroleum Engineering, NED University of Engineering & Technology, Karachi 75270, Pakistan; § School of Engineering, Edith Cowan University, 270 Joondalup Drive, Joondalup, WA 6027, Australia; ∥ Center for Integrative Petroleum Research (CIPR), College of Petroleum Engineering and Geosciences, King Fahd University of Petroleum and Minerals, Dhahran 31261 ,Saudi Arabia

## Abstract

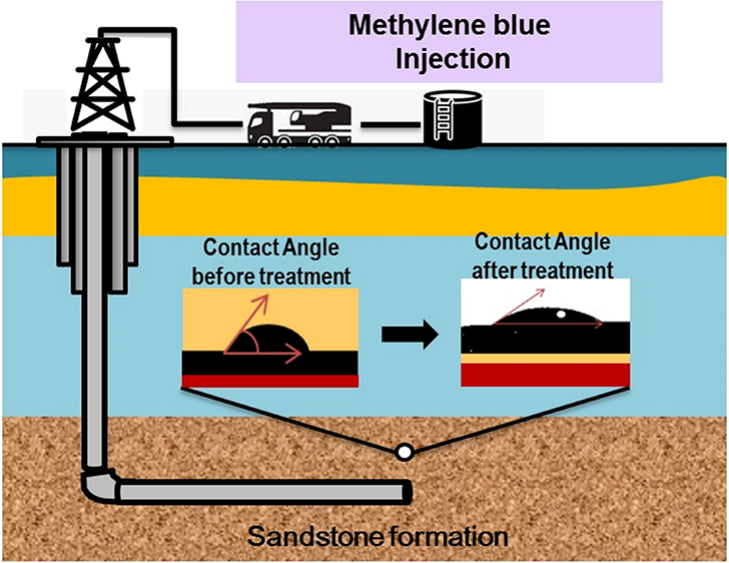

Significant amounts of hydrocarbon resources are left behind after primary and secondary recovery processes, necessitating the application of enhanced oil recovery (EOR) techniques for improving the recovery of trapped oil from subsurface formations. In this respect, the wettability of the rock is crucial in assessing the recovery and sweep efficiency of trapped oil. The subsurface reservoirs are inherently contaminated with organic acids, which renders them hydrophobic. Recent research has revealed the significant impacts of nanofluids, surfactants, and methyl orange on altering the wettability of organic-acid-contaminated subsurface formations into the water-wet state. This suggests that the toxic dye methylene blue (MB), which is presently disposed of in huge quantities and contaminates subsurface waters, could be used in EOR. However, the mechanisms behind hydrocarbon recovery using MB solution for attaining hydrophilic conditions are not fully understood. Therefore, the present work examines the impacts of MB on the wettability reversal of organic-acid-contaminated Khewra sandstone samples (obtained from the outcrop in the Potwar Basin, Pakistan) under the downhole temperature and pressure conditions. The sandstone samples are prepared by aging with 10^–2^ mol/L stearic acid and subsequently treated with various amounts of aqueous MB (10–100 mg/L) for 1 week. Contact angle measurements are then conducted under various physio-thermal conditions (0.1–20 MPa, 25–50 °C, and salinities of 0.1–0.3 M). The results indicate that the Khewra sandstone samples become hydrophobic in the presence of organic acid and under increased pressure, temperature, and salinity. However, the wettability changes from oil-wet to preferentially water-wet in the presence of various MB solutions, thus highlighting the favorable effects of MB on EOR from the Khewra sandstone formation. Moreover, the most significant change in wettability is observed for the Khewra sandstone sample that was aged using 100 mg/L MB. These results suggest that injecting MB into deep underground Khewra sandstone reservoirs may produce more residual hydrocarbons.

## 1 Introduction

The global energy demand is rising and projected to increase significantly in the upcoming years^1,2^ and may even increase by around 60% by 2040, primarily due to population growth, urbanization, and economic development in many countries.^3−5^ Crude oil is the principal source of energy worldwide, and the increasing number of depleted oil reservoirs (containing approximately 70% of the remaining crude oil) requires immediate attention.^6,7^ Hence, the development of innovative production enhancement strategies is essential in order to cope with the increasing energy demand and to optimize recovery from existing hydrocarbon fields.^8,9^ Several enhanced oil recovery (EOR) methods, such as chemical EOR and thermal EOR, are used to increase the recovery and sweep efficiency during water flooding and gas injection.^10−15^ In this respect, the wettability of the rock is an essential parameter for improving the trapped oil recovery and sweep efficiency.^16,17^ However, subsurface formations are naturally contaminated with organic acids, which renders them hydrophobic^18−23^ and leads to the early breakthrough of injected fluid, which leaves behind large volumes of hydrocarbons.^24,25^

Recent studies have demonstrated the wettability alteration of organic-acid-contaminated rocks by using nanofluids, surfactants, and certain chemicals such as methyl orange for EOR and gas (CO_2_ and H_2_) geo-storage purposes.^24,26−33^ The results have shown that these surface-active agents can favor chemical flooding and CO_2_ injection for EOR and CO_2_ geo-storage.^28−30,34,35^ Mechanistically, the presence of surfactants, nanoparticles, and methyl orange in the base fluid has a robust effect on altering the hydrophobic rock surface into a water-wet state.^36^ Similarly, CO_2_ flooding has also depicted improved oil recovery in many previous studies.^37,38^ In addition, changes in the oil viscosity and reduction of the interfacial tension (IFT) have contributed to the success of EOR projects.^39^

Methylene blue (MB) is a commonly used dye and is presently discharged in large volumes as a component of wastewater by textile and other industries.^40,41^ This wastewater contamination leads to environmental pollution and hazards to health.^42^ Although there are several techniques by which MB can be removed from wastewater, most of these are not economical, and managing the massive quantities of industrially produced wastewater is presently challenging.^42−47^ Most recently, Alhamad et al.^41^ reported that the treatment of organic-molecule-contaminated quartz with MB significantly reduced the contact angle (CA) and restored the initial hydrophilic state of the quartz for enhanced H_2_ geo-storage capacity. However, the CA measurement was conducted only for H_2_/brine systems, so that their results only assess the underground hydrogen storage potentials of MB-treated quartz. Meanwhile, the EOR potential of MB-treated sandstone formations remains largely unexplored.

The present study therefore examines the feasibility of using MB to modify the wettability of sandstone samples from the Khewra outcrop in the Potwar Basin, Pakistan, for EOR. The sandstone substrates are first aged in stearic acid (SA)/*n*-decane solution (10^–2^ mol/L) and then placed in various concentrations of aqueous MB (10, 30, 50, 80, and 100 mg/L). The wettability is then measured in air-brine and oil–water systems at various temperatures (25 and 50 °C), salinities (0.1, 0.3, and 0.5 M), and pressures (0.1, 5, 10, 15, and 20 MPa). The results of this study are expected to be beneficial for minimizing the environmental impacts of MB and enhancing the oil recovery of organic-acid-contaminated sandstone formations.

## 2 Geology of the Studied Area

**Figure 1:**
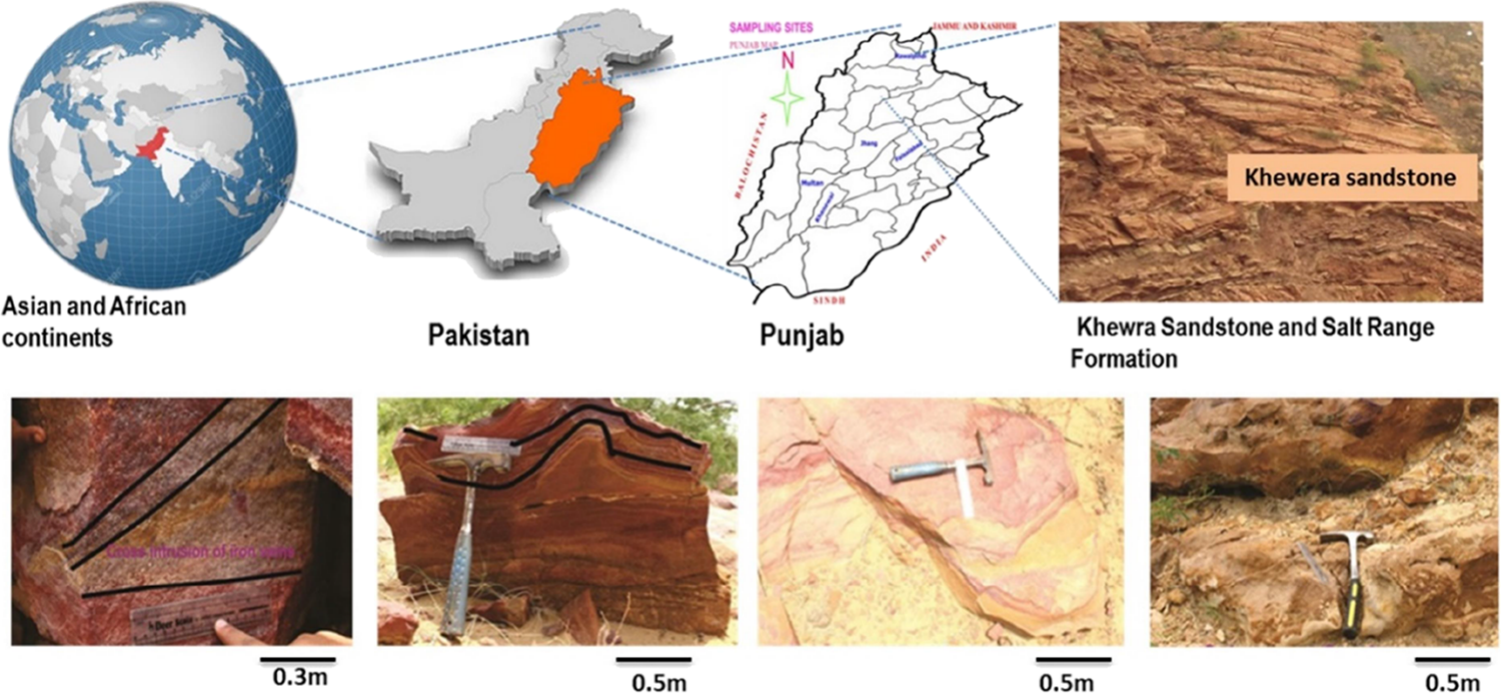
Geographic locations of Pakistan and the sample collection site in the Khewra sandstone formation.

**Figure 2:**
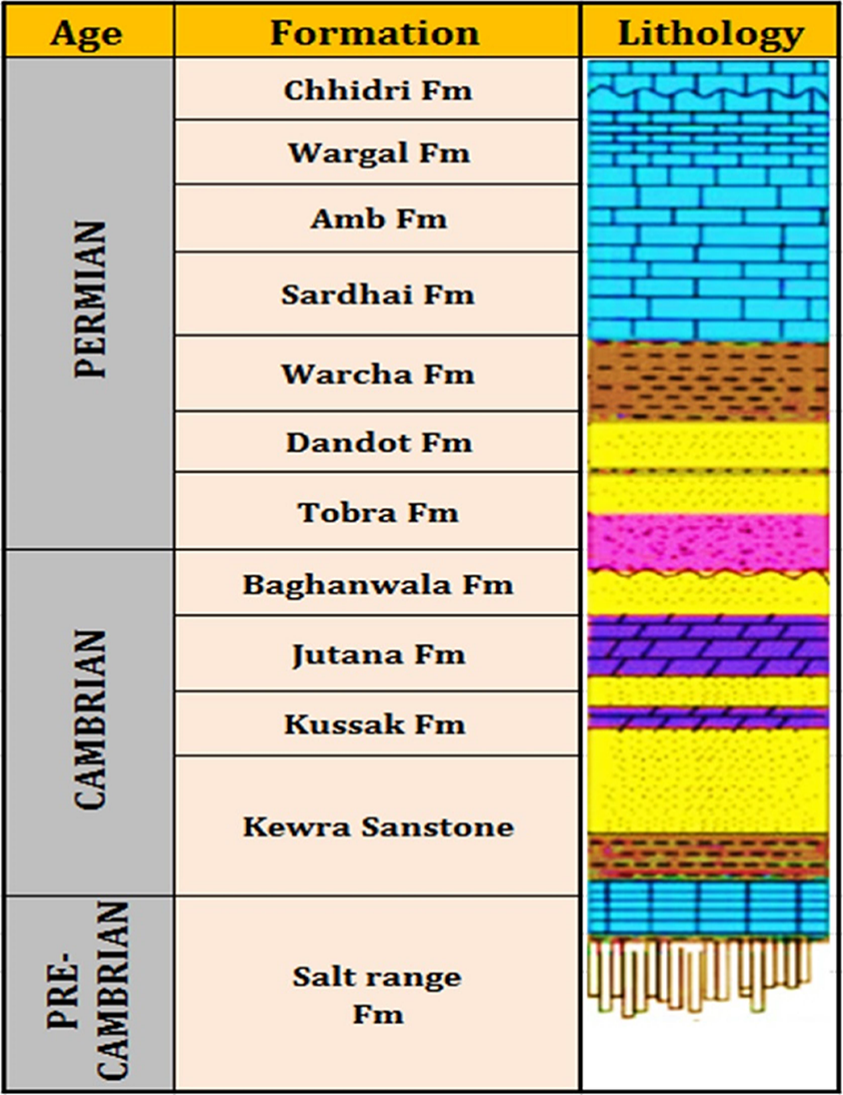
Generalized stratigraphic section of the salt range in the Potwar Basin, Pakistan.

The location of Pakistan and the sample collection site (the Khewra sandstone formation) are shown in Figure 1, and the general stratigraphy of the Potwar Basin is shown in Figure 2. Pakistan sedimentary basins are rich in hydrocarbon potential, covering an area of 873,000 km^2^ including most of eastern Pakistan and western part of India.^48^ The Potwar Basin in Pakistan is characterized as a complex sequence of sedimentary rocks deposited over millions of years. The geological sequence of the Potwar Basin can be divided into several distinct formations and layers, representing different geological periods and processes. The oldest clastic rocks of the Potwar Basin are found in the thick Khewra sandstones assemblage and were deposited millions of years ago during the Pre-Cambrian era.^49^ These rocks are typically crystalline and metamorphic.^50^ However, the region’s substantial geological features primarily consist of sandstones that were deposited during the Mesozoic era, specifically during the Jurassic period. The Khewra sandstone is a sedimentary rock composed mainly of sand-sized mineral particles or rock fragments, probably due to the accumulation and cementation of sand grains over millions of years.^49^ These geological formations possess significant hydrocarbon reserve potential,^51^ and the region around the Khewra sandstone formation is known for its rich mineral resources.

## 3 Materials and Methods

### 3.1 Materials

The study samples were obtained from the Khewra sandstone formation of the Potwar Basin, Punjab, Pakistan, and represented a wide range of sedimentary environments and postdepositional conditions (Figures 1 and 2). To assess the wetting characteristics, the samples were aged in SA (Sigma-Aldrich, purity ≥99.999 mol %) as a geologically representative organic acid, and MB (Sigma-Aldrich) was used as a wettability modifier (Section 3.2). The brine solutions with various desired concentrations (0.1–0.3 M) were prepared by dissolving sodium chloride (NaCl, purity 99.999 mol %; Chemlabs) in deionized water. N-Decane (from Sigma-Aldrich) was used as a nonwetting phase during the CA measurements, and ultrapure nitrogen (≥99.999 mol %) was used to clean the organic-contaminated Khewra sandstone substrates.

### 3.2 Cleaning and Aging Procedures

**Figure 3:**
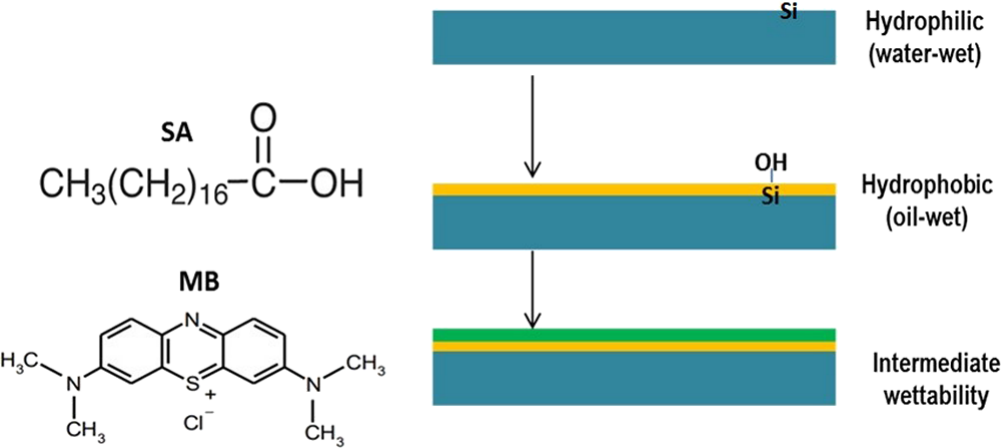
Chemical structures of SA and MB (left) and their effects on the sample surface (right). Modified with permission from ref (41). Copyright 2023, Authors and Elsevier.

Thin rectilinear sections (10 × 10 × 3 mm) of the Khewra sandstone were precisely machined from small cubes of about 1.52 × 1.40 × 0.50 cm in size and then polished with abrasive sandpaper (1000 to 400 mesh) to obtain a smooth surface before measuring the CA. In addition, to eliminate any surface contamination (e.g., deposits of organic matter), which might otherwise lead to substantial measurement errors, the sample surfaces were cleaned with deionized water, followed by drying and blowing with ultrapure nitrogen (≥99.999 mol %). The Khewra sandstone substrates were then placed in an oven for 2 h to remove any in situ water. After this, the Khewra sandstone substrates were ionized with a 2 wt % NaCl solution, while the pH was held at 4 by dropwise addition of aqueous HCl, followed by aging with SA/*n*-decane solution (10^–2^ mol/L) for 7 days at 50 °C. This process increases the adsorption potential of the sample surface and mimicks the natural geological conditions under which the rock is exposed to organic compounds for millions of years.^18,19,52−54^ Finally, the SA-aged Khewra sandstone substrates were aged in various concentrations of MB solution (10–100 mg/L) to examine the wettability reversal for enhanced hydrocarbon potential. The mechanisms of surface treatment with SA and MB are shown schematically in Figure 3.

### 3.3 Contact Angle Measurement

**Figure 4:**
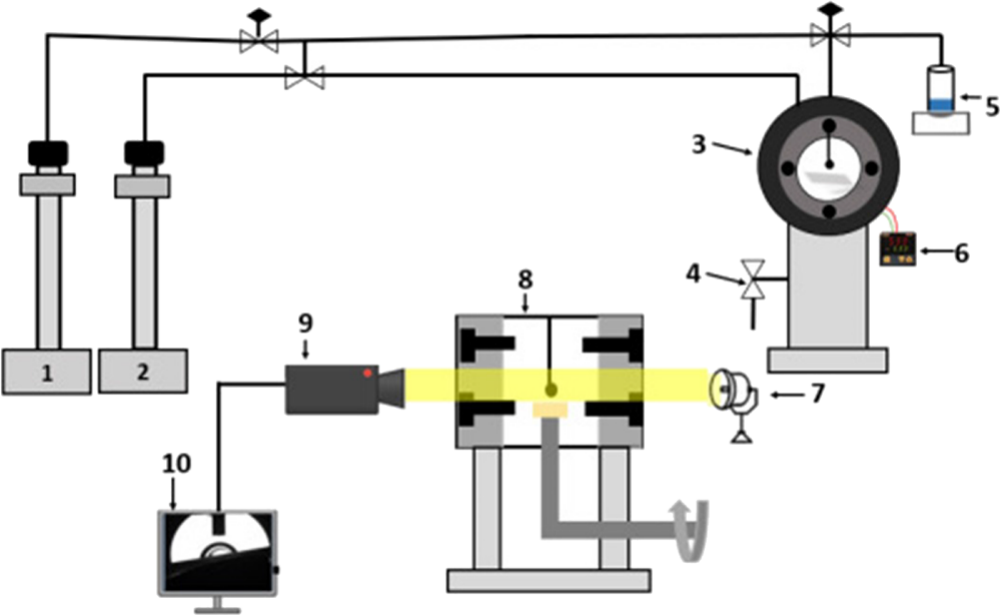
Schematic diagram of the experimental setup for the CA measurements: (1) ISCO pump for brine injection; (2) ISCO pump for *n*-decane injection; (3) IFT cell (face-on view); (4) relieve valve; (5) brine solution; (6) heating controller; (7) lamp; (8) IFT cell (side view); (9) video camera; and (10) computer with ImageJ software. Reprinted with permission from ref (24). Copyright 2022, Authors and Elsevier.

Contact Angle (CA) measurement is an effective technique for studying the wettability of solid surfaces.^55−58^ It is a quantitative method that provides direct information regarding the wetting characteristics of the rock.^1^ The experimental setup for the static CA measurement is schematically shown in Figure 4. First, the samples are placed on a flat surface in the optical cell (3, 8), and then the *n*-decane solution (as a representative hydrocarbon) is injected via a high-precision ISCO syringe pump (2) (Teledyne ISCO D-260; pressure accuracy = 0.01%) to fill the IFT cell at the required pressure and temperature. A brine droplet with an average size of 6.2 μL ± 0.6 μL is then injected through a precise needle controlled by another ISCO pump (1). The procedure is recorded using a high-magnification video camera (9), and the CA images are extracted and analyzed by using the ImageJ software (10) to measure the tangent angles. In the present study, CA measurements were performed at both 25 and 50 °C under pressures of 0.1 to 20 MPa. Each CA measurement was repeated three times to obtain the mean value with an error of only ±3°.

### 3.4 Characterization

The Khewra sandstone samples were examined via atomic force microscopy (AFM; Nanosurf, Controller C3000, and Flex-Axiom) over an area of 10 × 10 μm^2^ to determine the surface roughness. Field emission scanning electron microscopy (FESEM; Oxford Instruments) was used to determine the surface morphology. The functional groups resulting from MB and SA adsorption were identified via Fourier transform infrared (FTIR) spectroscopy (PerkinElmer two, USA) in the range of 400–4000 cm^–1^.

## 4 Results and Discussion

### 4.1 Surface Characterization

**Figure 5:**
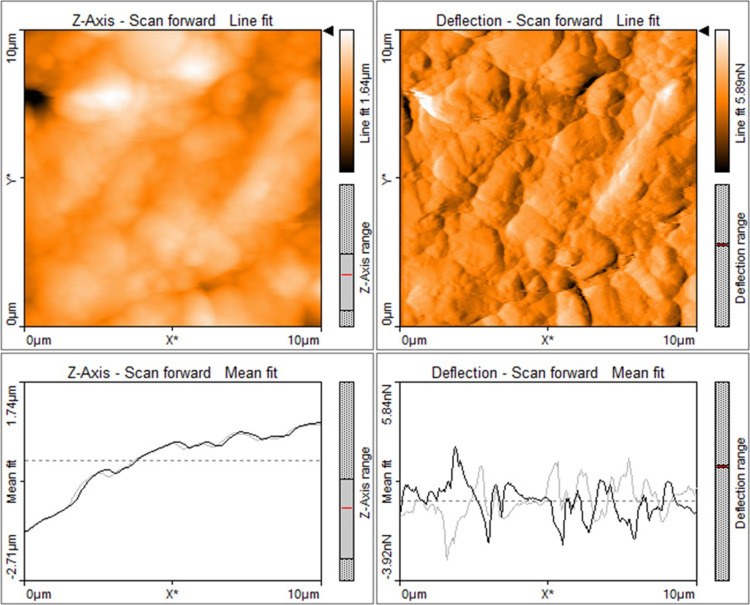
AFM images (top) and surface profiles (bottom) of the pure Khewra sandstone sample.

The surface roughness can significantly influence the measured wettability of the rock.^59−62^ Studies have shown that an increase in surface roughness leads to a decrease in the CA because water is retained in the grooves of the rough surface, thus resulting in increased hydrophilicity.^60,62,63^ However, if the surface roughness is less than 1 μm, then the CA measurement is not significantly affected.^60,64^ Therefore, the surfaces of the Khewra sandstone substrates are revealed by the AFM image in Figure 5. Here, the surface of the pristine sample exhibits a roughness of around 234 nm, whereas the sample that was treated with SA and MB exhibits a nonuniform layer with an increased surface roughness of 345–476 nm.

**Figure 6:**
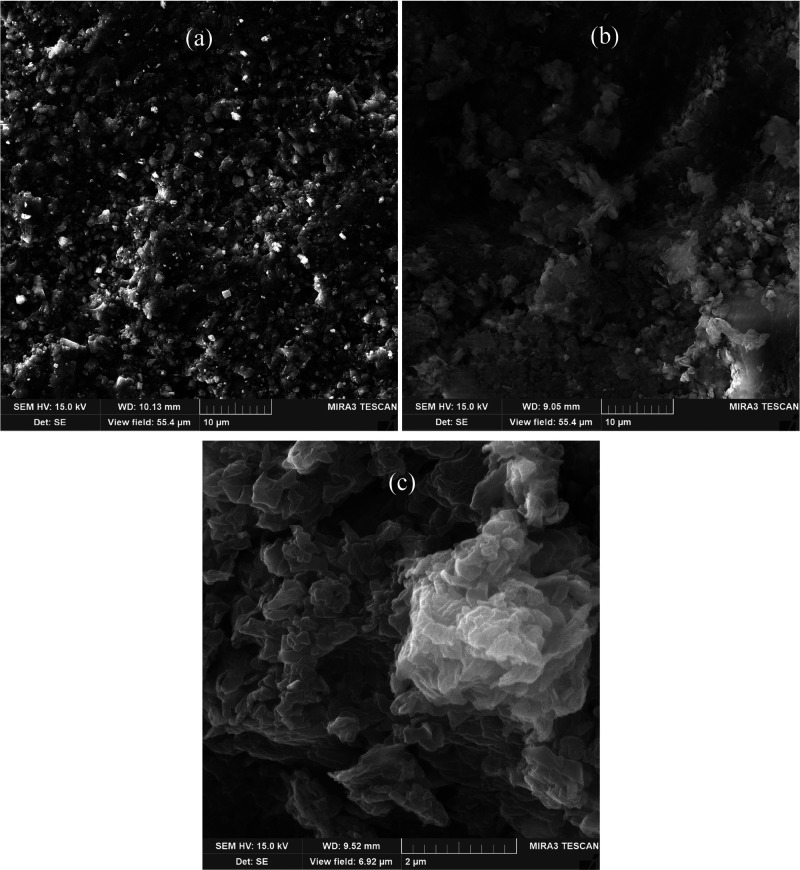
FESEM images of the Khewra sandstone samples (a) before and (b, c) after treatment with (b) SA and (c) MB.

The FESEM images of the samples before and after treatment with SA and MB are presented in Figure 6. Here, the untreated Khewra sandstone sample exhibits a smooth, rocky texture (Figure 6a), whereas the SA- and MB-treated samples each exhibit distinct textures due to surface modification (Figure 6b,c). This demonstrates the irreversible adsorption of both SA and MB on the sample surfaces,^41,65,66^ which is responsible for altering the wettability.^31,54^

**Figure 7:**
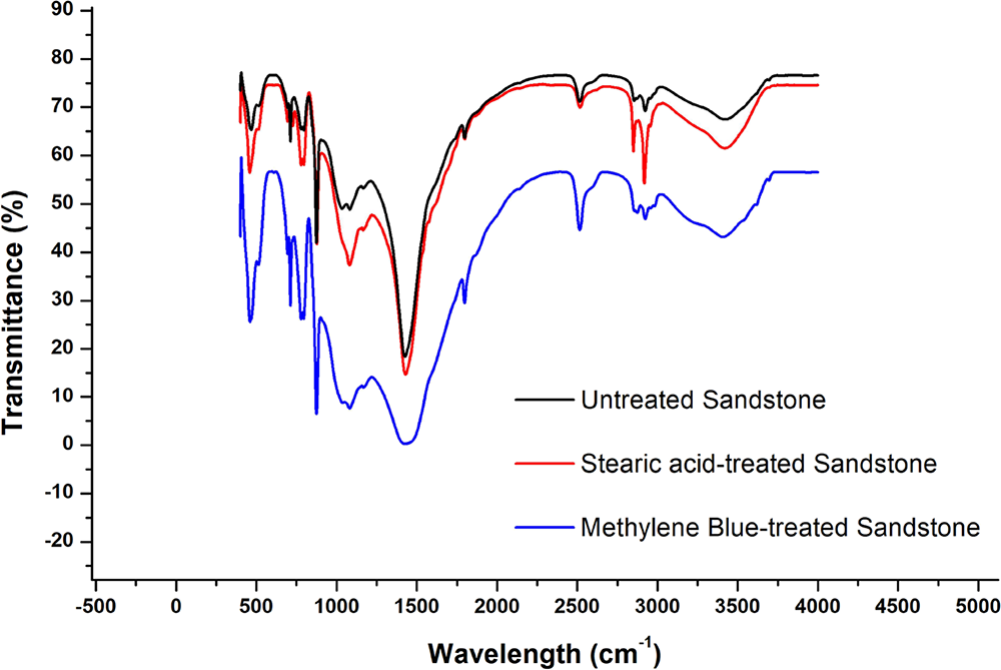
FTIR spectra of the Khewra sandstone samples before and after treatment with SA and MB.

The FTIR spectra of the Khewra sandstone samples before (black) and after treatment with SA (red) and MB (blue) are presented in Figure 7. Here, the samples are seen to be composed primarily of quartz, with the corresponding Si–O peaks appearing at 989, 897, 758, and 525 cm^–1^. The absorption band at 837 and 539 cm^–1^ corresponds to the bending and stretching vibration of the SiO_2_ group. However, the intensities of the peaks at 3000–3700 cm^–1^ are seen to decrease after the SA and MB treatments, which is attributed to the formation of hydrogen and oxygen bonds. The resulting Si–OH groups are responsible for the observed surface modifications.^26,41,67^

### 4.2 Effect of Methylene Blue on the Wettability of the Khewra Sandstone Samples

**Figure 8:**
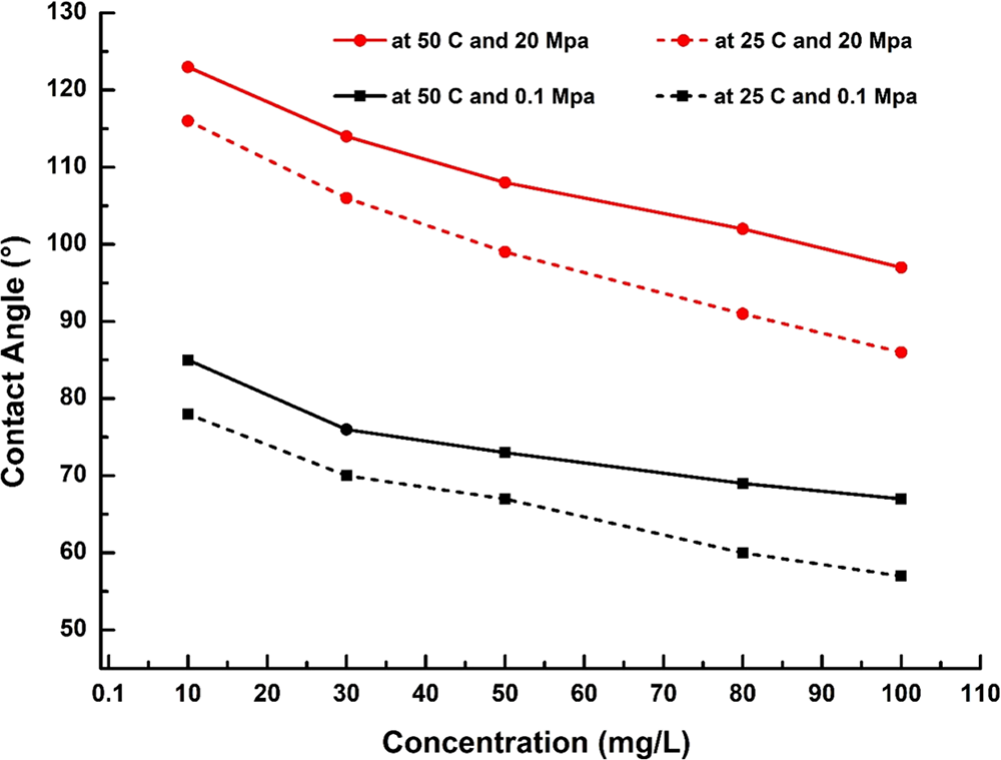
Effects of various concentrations of MB on the wetting behavior of SA-aged Khewra sandstone samples at various temperatures and pressures.

The effects of various MB concentrations on the wettability of the SA-aged (10^–2^ mol/L) Khewra sandstone samples with 0.3 molar salinity at various temperatures and pressures are presented in Figure 8. Here, the CA values exhibit a general decrease with an increase in the MB concentration. For instance, at 25 °C and 0.1 MPa (black dashed line), the CA decreases from 78 to 57° as the MB concentration is increased from 10 to 100 mg/L MB. Similarly, at 50 °C and 20 MPa (red solid line), the CA decreases from 123 to 97° as the MB concentration is increased from 10 to 100 mg/L MB. These results indicate that the SA-aged sandstone tends to become more hydrophilic when treated with increasing concentrations of MB.

**Figure 9:**
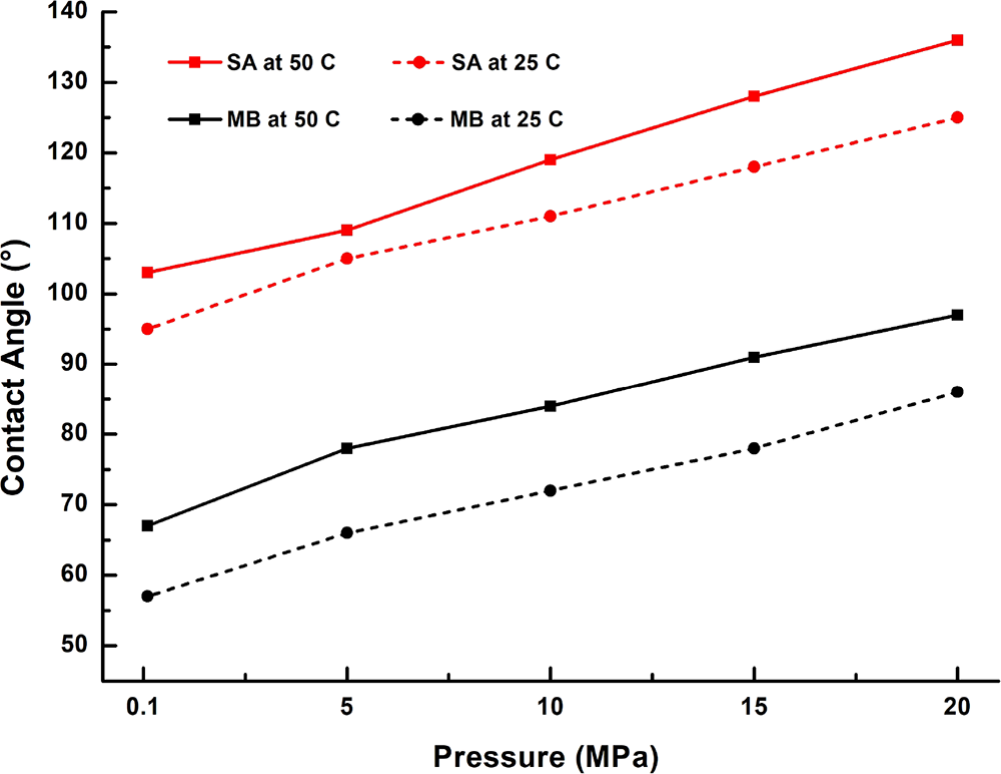
Effect of varying pressures on the CA values of the SA- and MB-aged Khewra sandstone samples at temperatures of 25 and 50 °C.

Before the MB treatment, the CA values of the SA-aged Khewra sandstone samples at 25 °C are 95 and 125° at pressures of 0.1 and 20 MPa, respectively (Figure 9). At a higher temperature of 50 °C, the corresponding CA values are 103 and 136°, respectively. Thus, the surfaces of Khewra sandstone outcrop substrates became hydrophobic in the presence of organic acid, in agreement with previous studies.^26−31,56,60^ However, as demonstrated in Figure 8, the exposure of the organic acid-aged Khewra sandstone samples to various concentrations of MB results in the adsorption of MB onto the rock surface via van der Waals interactions.^68−70^ Similar reductions in the CA values of organic-acid-contaminated samples have been reported after treatment with increasing concentrations of nanofluids, surfactants, and methyl orange.^24,28−30,34,35,71^ For example, Ali et al.^28,29^ examined the influence of SiO_2_ and Al_2_O_3_ nanofluids on the wettability of organic acid-aged quartz and mica substrates and observed that the surface wettability was modified due to the adsorption of the nanofluids on the aged rock surfaces. Similarly, Alhamad et al.^24^ reported a considerable reduction in the CA of SA-contaminated quartz when the rock was treated with methyl orange.

### 4.3 Effects of Pressure and Temperature on the Wettability of the Khewra Sandstone in the Presence of Organic Acid and Methylene Blue

The effects of pressure variations on the wettabilities of the SA- (10^–2^ mol/L) and 100 mg/L MB-treated Khewra sandstone samples with 0.3 molar salinity at temperatures of 25 and 50 °C are presented in Figure 9. Here, the CA values are seen to increase with increasing pressure and temperature. However, the effect of pressure is much more profound for the SA-aged samples than for the MB-treated samples, as evidenced by the much steeper gradient of the solid red line (SA-aged, 50 °C) compared with the black solid line (MB-treated, 50 °C). The effects of temperature are also distinct for the SA-aged samples compared to that for the MB-treated ones. Thus, at a fixed temperature of 50 °C, the CA value of the SA-aged sample (red solid line) increases significantly from 109° to 136 °C as the pressure is increased from 5 to 20 MPa, whereas the CA of the MB-treated sample (black solid line) increases more slightly from 78 to 97° over the same range of pressures. Meanwhile, at a constant pressure of 20 MPa, the CA value of the SA-aged sample decreases from 136 to 125° as the temperature decreases from 50 °C (red solid line) to 25 °C (red dashed line), while the CA of the MB-treated sample decreases from 97 to 86° as the temperature decreases from 50 °C (black solid line) to 25 °C (black dashed line), respectively. Thus, it can be concluded that the oil-wet Khewra sandstone samples change from oil-wet when aged in SA to intermediate-wet when aged in MB at a sufficiently high temperature.

The dependence of temperature on rock wetting behaviors and the attendant effects on oil recovery from sandstone formations have been investigated in previous studies.^72−77^ Although some reported results have been quite contradictory,^24,78^ the common trend regarding the impact of temperature on rock wettability is that increasing the temperature enhances the capacity of the trapped oil to flow compared to that of the water; hence, the water-wetness of the rock decreases at elevated temperatures. Moreover, an increase in temperature reduces the oil–water IFT and decreases the viscosity of trapped oil, thus resulting in an overall increase in oil recovery from sandstone formations.^79−81^

Similarly, an increased pressure results in increased CAs in the SA- and MB-aged Khewra sandstone samples. However, the degree of change depends on the specific temperature and surface modification conditions, as noted above. The observed change in wettability due to the increase in pressure is related to the intermolecular attraction between liquid molecules and the rock surface, thus making the Khewra sandstone samples more hydrophobic.^82,83^

### 4.4 Effect of Salinity on the Wettability of the Khewra Sandstone Samples in the Presence of Organic Acid and Methylene Blue

**Figure 10:**
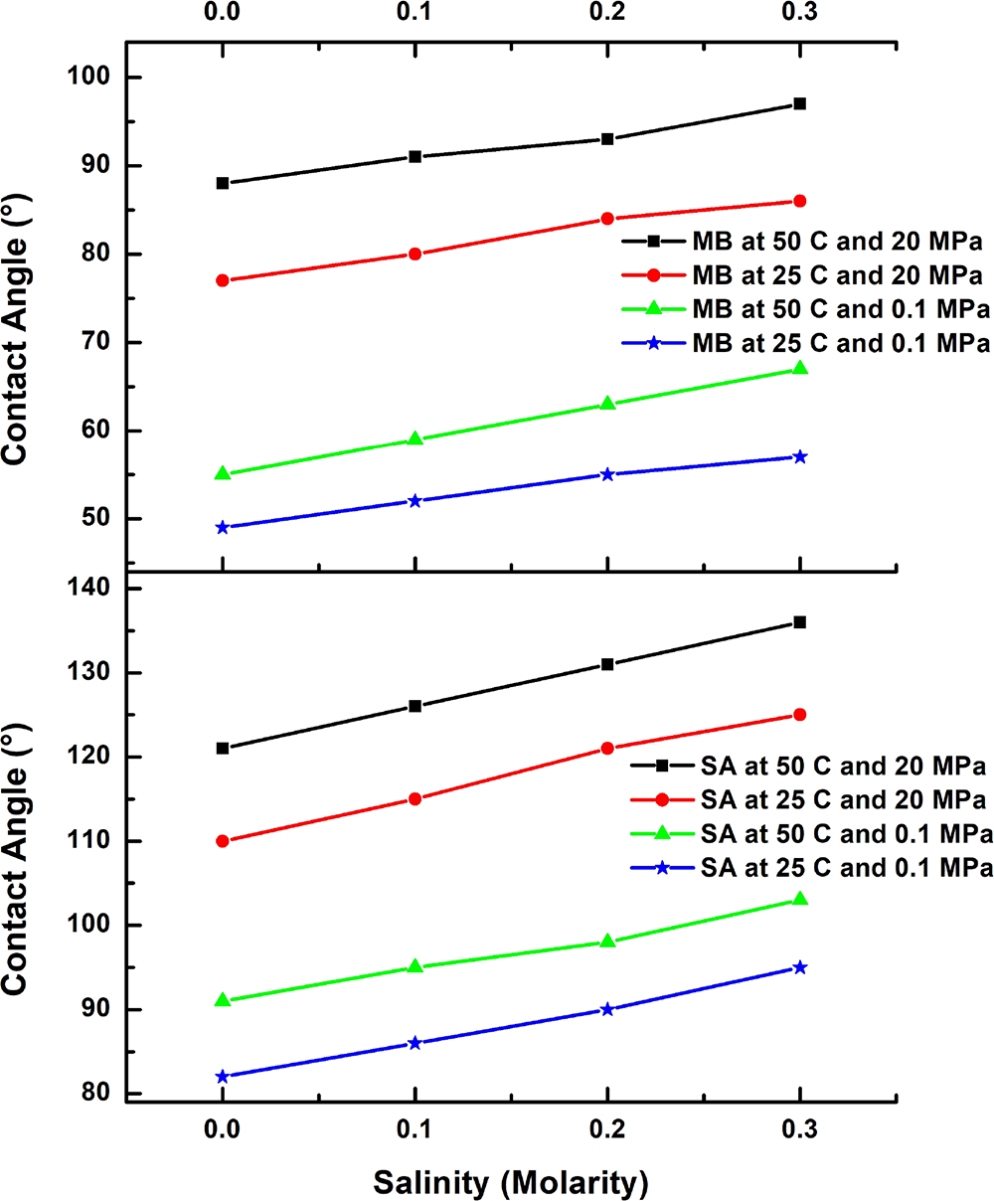
Effects of salinity on the CA values of the SA- and MB-aged Khewra sandstone samples at various pressures and temperatures.

The impacts of brine salinity on the wettabilities of rock surfaces have been emphasized in previous research.^60,84−87^ It is well-known that varying the concentration and type of reservoir brine significantly affects the wetting characteristics of the rock surface in the oil/brine environment.^24,88,89^ Therefore, the effects of increasing salinity (0.0 to 0.3 M) on the wettabilities of the SA- (10^–2^ mol/L) and 100 mg/L MB-aged Khewra sandstone samples are revealed by the CA measurements in Figure 10. Here, it can be seen that the CA values increase with the increase in salt concentration, which is consistent with the results of previous studies.^24,85,87,90,91^ However, the degree of change in the CA value is lower in the MB-aged samples than in the SA-aged ones. This confirms the effectiveness of MB in decreasing the hydrophobicity of the SA-contaminated Khewra sandstone samples, thereby increasing their hydrocarbon potential. For instance, at 50 °C and 20 MPa, the CA of the MB-aged Khewra sandstone increases from 88 to 97° (a difference of 9°) as the brine salinity is increased from 0.0 to 0.3 M, while the CA of the SA-aged sandstone increases from 121 to 136° (a difference of 15 units) under the same conditions.

The effect of increasing salinity on the wettability of the rock surface can be attributed to the screening effect due to the brine-induced surface charge,^27^ which may become positive at higher salt concentrations, thereby nullifying the original negative surface charge of the sandstone.^90,92^ This, in turn, decreases the interactions between the rock surface and water, thereby increasing the attraction between the oil and the rock surface to favor the oil-wet behavior. For example, Pan et al.^93^ reported that the zeta potential of a shale sample surface increased when CaCl_2_ and NaCl were introduced into the system due to the surface adsorption of divalent ions, thus resulting in a positive charge. Similarly, Kaya and Yukselen^94^ reported an increase in the zeta potential of quartz when the salt concentration was increased.

## 5 Conclusions

Wettability is an essential property of subsurface reservoirs, influencing the fluid flow dynamics, displacement, and hydrocarbon recovery rate.^1,16,95−97^ Hydrocarbon reservoirs are inherently hydrophobic due to dissolved organics.^98,99^ Meanwhile, MB dye has been extensively used in various industries, including paper and textiles. It is typically discharged in massive quantities as industrial wastewater, which contaminates the subsurface water and poses a hazard to human health and the environment.^40,41^ Therefore, this study examined the feasibility of using MB to modify the wettability of organic-acid-contaminated Khewra sandstone samples obtained from the Potwar Basin, Pakistan, with the simultaneous aims of enhancing the hydrocarbon recovery and minimizing the environmental impact of MB by injecting it into hydrocarbon-producing reservoirs.

The results demonstrated that the CA values of the Khewra sandstone samples that were aged with 10^–2^ mol/L SA increased as the temperature (from 25 to 50 °C), pressure (from 0.1 to 20 MPa), and salinity (0.1 to 0.3 M) increased, thereby attaining completely hydrophobic (oil-wet) conditions. However, under similar reservoir conditions, the SA-aged Khewra sandstone samples were modified from their initial oil-wet state to an intermediate-wet state as the concentration of MB was increased from 10 to 100 mg/L. Moreover, the maximum reduction in CA value was achieved in the presence of 100 mg/L MB. These results suggest that treating organic-acid-contaminated Khewra sandstones with MB could considerably promote their water wetness, thereby improving the oil recovery from this and similar formations.
